# Prediction of multiple pH compartments by deep learning in magnetic resonance spectroscopy with hyperpolarized ^13^C-labelled zymonic acid

**DOI:** 10.1186/s13550-022-00894-y

**Published:** 2022-04-23

**Authors:** Wai-Yan Ryana Fok, Martin Grashei, Jason G. Skinner, Bjoern H. Menze, Franz Schilling

**Affiliations:** 1 Department of Informatics, Technical University of Munich, 85748 Garching, Germany; 2 Department of Nuclear Medicine, TUM School of Medicine, Klinikum Rechts der Isar, Technical University of Munich, 81675 Munich, Germany; 3 Munich Institute of Biomedical Engineering, Technical University of Munich, 85748 Garching, Germany

**Keywords:** Deep learning, Convolutional neural network, pH, Hyperpolarized 13C MRSI

## Abstract

**Background:**

Hyperpolarization enhances the sensitivity of nuclear magnetic resonance experiments by between four and five orders of magnitude. Several hyperpolarized sensor molecules have been introduced that enable high sensitivity detection of metabolism and physiological parameters. However, hyperpolarized magnetic resonance spectroscopy imaging (MRSI) often suffers from poor signal-to-noise ratio and spectral analysis is complicated by peak overlap. Here, we study measurements of extracellular pH (pH_e_) by hyperpolarized zymonic acid, where multiple pH_e_ compartments, such as those observed in healthy kidney or other heterogeneous tissue, result in a cluster of spectrally overlapping peaks, which is hard to resolve with conventional spectroscopy analysis routines.

**Methods:**

We investigate whether deep learning methods can yield improved pH_e_ prediction in hyperpolarized zymonic acid spectra of multiple pH_e_ compartments compared to conventional line fitting. As hyperpolarized ^13^C-MRSI data sets are often small, a convolutional neural network (CNN) and a multilayer perceptron (MLP) were trained with either a synthetic or a mixed (synthetic and augmented) data set of acquisitions from the kidneys of healthy mice.

**Results:**

Comparing the networks’ performances compartment-wise on a synthetic test data set and eight real kidney data shows superior performance of CNN compared to MLP and equal or superior performance compared to conventional line fitting. For correct prediction of real kidney pH_e_ values, training with a mixed data set containing only 0.5% real data shows a large improvement compared to training with synthetic data only. Using a manual segmentation approach, pH maps of kidney compartments can be improved by neural network predictions for voxels including three pH compartments.

**Conclusion:**

The results of this study indicate that CNNs offer a reliable, accurate, fast and non-interactive method for analysis of hyperpolarized ^13^C MRS and MRSI data, where low amounts of acquired data can be complemented to achieve suitable network training.

## Introduction

In living species, extracellular pH (pH_e_) is an important physiological parameter that is tightly regulated by intrinsic buffer systems. Locally, deviations from the systemic pH are often caused by pathologies, such as cancer, inflammation, infection, ischemia, renal failure or pulmonary disease [1–3]. Since pH_e_ can play a critical role in disease progression [4] and can influence therapeutic success [5], many efforts have been undertaken to develop a quantitative non-invasive pH imaging technique [3, 4, 6]. However, there is no clinical routine method available for spatial quantification of pH_e_, rendering it still an important target in biomedical imaging.

Magnetic resonance-based pH imaging methods offer high spatial resolution without limitations on the penetration depth and without involving ionizing radiation. In addition, conventional ^1^H MRI offers high anatomical soft tissue contrast that can be overlaid on top of pH images. MRI-based pH_e_ imaging techniques that have been applied in vivo require the use of exogenous molecules and rely either on their pH-dependent chemical exchange saturation transfer (CEST) or on their pH dependence of chemical shifts [1]. Utilizing endogenous molecules, the intracellular pH (pH_i_) can be measured by pH_i_-dependent proton exchange from amide groups of intracellular proteins [4].

Magnetic resonance-based detection of biochemical and physicochemical quantities by exogenous molecules was revolutionized by dissolution dynamic nuclear polarization (DNP) which lifts nuclear spin polarization to a so-called hyperpolarized state leading to a sensitivity gain of more than four orders of magnitude [7]. Hyperpolarized [1-^13^C]pyruvic acid is currently being used in clinical studies to examine its use for metabolic imaging of cancer, as well as in the brain and the heart [8–10]. Several pH-sensitive molecules have been hyperpolarized and been used for in vitro pH mapping including ^13^C, ^15^N, ^31^P, ^89^Y and ^129^Xe spin-1/2 nuclei [11]. Only two of those have so far been applied for pH imaging in vivo: hyperpolarized ^13^C-labelled bicarbonate [3, 12] and hyperpolarized [1,5-^13^C_2_]zymonic acid (ZA) [13] as well as its deuterated variant [1,5-^13^C_2_,3,6,6,6-D_4_]zymonic acid (ZA_d_) [14].

With hyperpolarized bicarbonate, pH_e_ is being determined by the signal intensity ratio of the CO_2_ and HCO_3_^−^ peaks, while the pH_e_ determination with ZA works via spectral analysis of the peak position, i.e. the chemical shifts. Chemical shift-based pH_e_ detection offers the unique advantage compared to intensity-based pH detection that multiple pH_e_ compartments within one imaging voxel can be resolved if their spectral peaks are separable, e.g. for resolving different pH_e_ compartments in the kidney [13]. For intensity-based pH_e_ detection, on the other hand, multiple pH compartments within one imaging voxel result in one signal intensity ratio, allowing only the determination of an average voxel pH. The concept of chemical shift-based detection of quantitative physiological measures using hyperpolarized magnetic resonance sensors has, besides for detection of pH_e_, also been used to quantify zinc [15], calcium/magnesium/iron ions [16], temperature [17], or ligand-receptor interactions [18].

Quantification of these measurements with hyperpolarized NMR sensors is done via analysis of the peak positions of the respective molecular sensors. Typically, the NMR spectra and all respective peaks are fitted via an optimization procedure giving the peak positions and amplitudes. However, such line-fitting procedures are error-prone in cases of low signal-to-noise ratio (SNR) and peak overlap, e.g. for multiple pH_e_ compartments within the kidneys [13]. In recent years, deep learning has shown its potential for magnetic resonance spectroscopy (MRS) and magnetic resonance spectroscopic imaging (MRSI) data in several applications to improve analysis of noisy data with interfering signals [19, 20]. Among these, artificial neural networks (ANN) demonstrated their value for spectroscopy analysis in medicine by classifying lung cancer tissue based on ^1^H MRS [21] or denoising of brain ^1^H MRS [22]. Furthermore, it was shown that convolutional neural networks (CNN) and multilayer perceptrons (MLP) can be trained to classify specific chemical compounds in various spectroscopy data sets [23, 24]. Nevertheless, we hypothesize that there is an advantage in applying a CNN for spectral analysis, as this class of network is invariant under frequency shifts of the entire spectrum which can be caused by B_0_ inhomogeneities.

We also hypothesize that transfer learning with real mice kidney data could improve the performance for our deep learning model. Transfer learning and domain adaptation have been used to adapt the model trained by one data distribution to the target data domain [25, 26], especially when the target domain data is limited [27]. Our target domain data, ^13^C-labelled zymonic acid kidney spectra, are by definition of the animal study and experimental efforts limited in size. Only one or two PRESS spectra or one CSI data set, still containing only a few single voxel spectra from kidneys, can be obtained from a single imaging experiment.

In this work, we investigate whether deep learning can improve the prediction of multiple pH_e_ compartments from magnetic resonance data using hyperpolarized ZA. For this task, we evaluate the performance of a CNN compared to a MLP as well as to conventional line fitting on both a single type of data (synthetic) and real data adaptation (a mix of real and synthetic data). For deep learning evaluation, both real data using line fitting as a gold-standard for evaluation of pH_e_ compartments as well as synthetic data with known pH_e_ compartments are used.

## Methods

### Neural network architecture

**Fig. 1:**
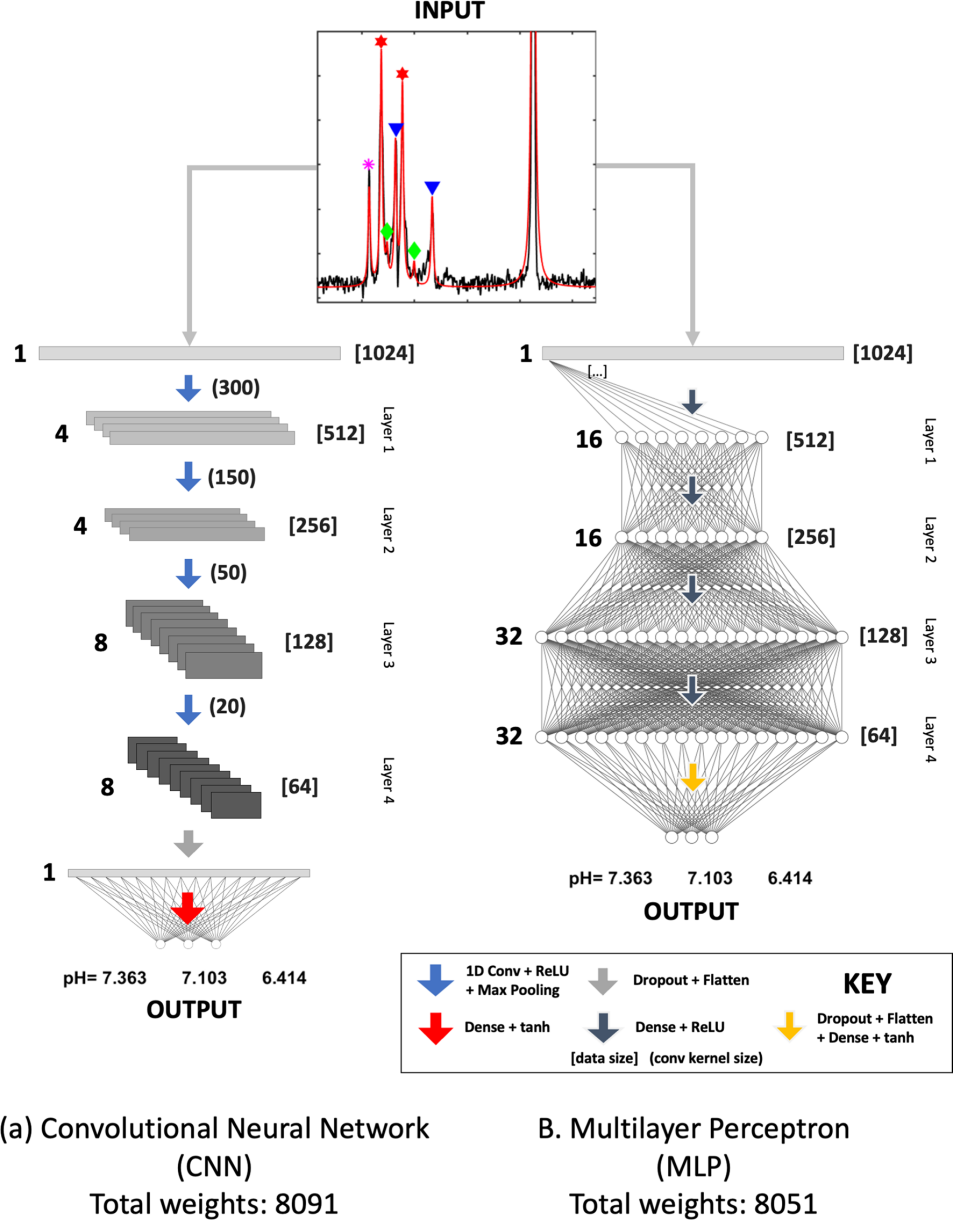
Schematic diagram of the neural network architecture for the **a** convolutional neural network (CNN) and the **b** multilayer perceptron (MLP). Both the proposed CNN and MLP consist of 4 feature extraction layers, for which they learnt a mapping between the input spectra and multiple pH_e_ compartments. To compare the performance of CNN and MLP in correctly predicting pH_e_ compartments, the architectures were set to a similar number of weights, ≈ 8000. The length of the spectrum or feature maps, which are used as the input to each next convolutional or dense layer, are shown in the square brackets. In CNN, the lengths are scaled logarithmically. CNN: The number of filters is 4, 4, 8, and 8. The sizes of the convolutional kernel are shown in the round brackets. MLP: The number of neurons is 16, 16, 32, and 32. Dense layers are represented with half (MLP) or quarter (CNN) the number of nodes, except for output layers

We implemented a multi-output regression convolutional neural network (CNN) and a multilayer perceptron (MLP), as shown in Fig. 1. The neural networks learned to map nuclear magnetic resonance spectra to specific pH_e_ values of a specific number of distinct pH_e_ compartments, of which were three for our specific case of in vivo kidney data of healthy mice.

The proposed CNN (Fig. 1a) consists of 4 sequential hidden layers for feature extraction. Each layer consists of a 1D convolutional kernel, a rectified linear unit (ReLU) activation function, and max pooling. The input into each layer was first convolved with the sliding kernel with stride of 1 and with length of 300, 150, 50 and 20. The initial weight (kernel) was a random value drawn from a truncated normal distribution. The convolutional kernel length decreased along the layers for extracting sub-regional features [28] as the input was downsampled from 1024 to 512, 256, 128 and 64 due to max pooling. The number of filters for feature extraction increased from 4, 4, to 8 and 8. ReLU activation functions were used in each layer to provide sparsity and thus robustness to small changes in input such as noise [29], as noise is inevitably present within the acquired spectra. The pooling layer was used to reduce the tensor size which could potentially merge semantically similar features [28].

Dropout was applied in the last feature extraction layer as a regularization to prevent over-fitting [30], for which it randomly dropped out 10% of the weights during training. Before the output layer, these feature maps were then flattened and passed to a dense layer with hyperbolic tangent activation function [31], and the outputs were then mapped into the target pH_e_ range 6.32 to 7.44.

The proposed MLP (Fig. 1b) consists of 4 fully connected dense layers, followed by max pooling. Each dense layer has a filter size 16, 16, 32 and 32, for which the output of each node is connected to all of the input nodes in the next layer. To compare their performance in mapping pH_e_ compartments, the architecture of CNN and MLP were designed such that the number of weights in both neural networks were similar, at approximately 8000.

### Modelling of NMR pH_e_ spectra

Relative to the urea peak position, the chemical shifts ZA_5_ and ZA_1_ can be described as a function of pH_e_ by the following scaled logistic function [13]:

1 $${\text{ZA}}_{i} \left( {{\text{pH}}_{{\text{e}}} } \right) = {\text{ZA}}_{i,\min } + \frac{{\delta_{i} }}{{1 + 10^{{\left( {{\text{pK}}_{{\text{a}}} - {\text{pH}}_{{\text{e}}} } \right)}} }}$$

where ZA_5,min_ = 12.57 ppm, ZA_1,min_ = 8.52 ppm, *δ*_1_ = 2.57 ppm, *δ*_5_ = 5.13 ppm and pK_a_ = 6.90 [13]. Using a Lorentzian peak model, the spectrum can be described by the following equation:

2 $$x\left( f \right) = \mathop \sum \nolimits_{i}^{N} \frac{{a_{i} \cdot w_{i}^{2} }}{{w_{i}^{2} + \left( {x + {\text{ZA}}_{i} \left( {{\text{pH}}_{{\text{e}}} } \right)} \right)^{2} }}$$

where *x*(*f*) is the spectrum, *N* is the number of peaks (in our case *n* = 7; 6 zymonic acid peaks and one urea peak), $a_{i}$ is the peak amplitude, $w_{i}$ is the full width at half maximum, and ZA_*i*_(pH_e_) is the corresponding chemical shifts of ZA_5_ and ZA_1_ peaks found by Eq. 1. For the ^13^C-urea peak, ZA_*i*_(pH_e_) is set to zero.

### Hyperpolarized ^13^C magnetic resonance spectroscopy

#### Hyperpolarization

27 mg [1,5-^13^C_2_,3,6,6,6-D_4_]zymonic acid [14], 1.7 mg Ox063 trityl radical (GE Healthcare, Chicago, IL, USA) and 24 µL DMSO were vortexed for 35 min. The mixture was added to a DNP sample cup and frozen in liquid nitrogen. 25 µL of a sample, containing 10 M ^13^C-urea in glycerol, 30 mM Ox063 and 1.5 mM DOTAREM (Guerbet, Villepinte, France) was subsequently added on top of the frozen layer and also frozen in liquid nitrogen. The sample cup was then polarized at 1.2 K for three hours by irradiation with a microwave frequency of 94.169 GHz and a power of 100 mW using a HyperSense® DNP Polarizer (Oxford Instruments, Abingdon, UK). Dissolution was performed with 2.99 ml D_2_O containing 80 mM TRIS, 0.3 mM EDTA and 50 mM NaOH, resulting in solutions containing 50 mM hyperpolarized zymonic acid and 100 mM urea with a pH 6.7 ± 0.4.

#### Hyperpolarized ^13^C-magnetic resonance spectroscopy

All experiments were performed on a horizontal bore small animal 7 T magnet MRI scanner (Agilent/GE) MR901 with Bruker AVANCE III HD electronics and a 31 mm ^1^H/^13^C volume resonator (RAPID Biomedical, Rimpar, Germany). Experiments were performed on seven healthy C57BL/6 mice (Charles River, Wilmington, MA, USA) in accordance with pertinent laws and regulations and approved by an ethical review board (Regierung von Oberbayern, Munich, Germany, Approval Number ROB-55.2-2532.Vet_02-17-177). Prior to imaging, animals were anaesthetized with 1.5–2.5% Isoflurane (v/v) in oxygen as a carrier gas, a tail vain catheter was placed and animals were positioned together with a 4.4 M [1-^13^C]lactate phantom for B_1_ calibration inside the magnet. Breathing rate (40 ± 7 min^−1^) and animal temperature (37.0 ± 0.6 °C) were monitored with an ECG trigger unit (RAPID Biomedical) and an MR-compatible temperature monitoring system Model 1030 (SA Instruments Inc, Stony Brook, New York, NY, USA) respectively. Kidneys were located using ^1^H RARE with FOV 32 × 32 mm^2^, slice thickness 1 mm, matrix size 128 × 128, repetition time 4000 ms, effective echo time 48 ms, RARE factor 12, 10 averages. Following manual B_1_ calibration using ^13^C-FID acquisitions with non-selective excitation by a 1 ms block pulse of varying RF power and fitting of the resulting signal vs. excitation power curves, the hyperpolarized solution was injected and ^13^C acquisitions started 5 s after end of injection. Single voxel ^13^C spectroscopy used PRESS on single kidneys with typical parameters: Total echo time 13.9 ms, total scan time 531 ms, excitation pulse flip angle 90°, refocusing pulse flip angle 180°, receive bandwidth 2000 Hz, 1024 points, voxel size 5 × 5 × 7 mm. Hyperpolarized ^13^C-MRSI was performed using FIDCSI with typical parameters: total scan time 14 s, FOV 28 × 24 mm^2^, slice thickness 5 mm, matrix size 14 × 12, repetition time 83.1 ms, flip angle 15°, receive bandwidth 3200 Hz, 256 spectral points. Overall, nine CSI data sets and six PRESS data sets were acquired and used for training and testing.

#### Data analysis and conventional line fitting

All data processing was performed in MatLab (The Mathworks Inc., Natick, MA, USA). For PRESS acquisitions, spectra were line-broadened by 5 Hz and phased manually. For CSI acquisitions, no line-broadening was applied, and magnitude spectra were averaged across both kidneys. C_1_- and C_5_-peaks of zymonic acid and of urea were identified by a standard automatic peak picking algorithm in MatLab for each pH_e_ compartment and selection was inspected manually. Peaks were fitted according to the model described in Eq. 2 where peak height, position relative to urea, and a uniform peak width for all compartments were fitted as free parameters using a built-in non-linear least squares algorithm. Second, the corresponding pH_e_ value was fitted from the relative zymonic acid peak distance to urea according to Eq. 1. Pairs of zymonic acid peaks were grouped into pH compartments and for each detected pH_e_ compartment, a mean pH_e_ was calculated which was weighted by the signal intensities from both the C_1_- and the C_5_-peaks. Common values for peak linewidths in Hz and ratios of signal amplitudes of the different kidney compartments were also extracted for generation of synthetic spectra for the training of the neural networks.

### Evaluation and training of the neural networks

#### Data sets

##### Spectra synthetization

**Fig. 2:**
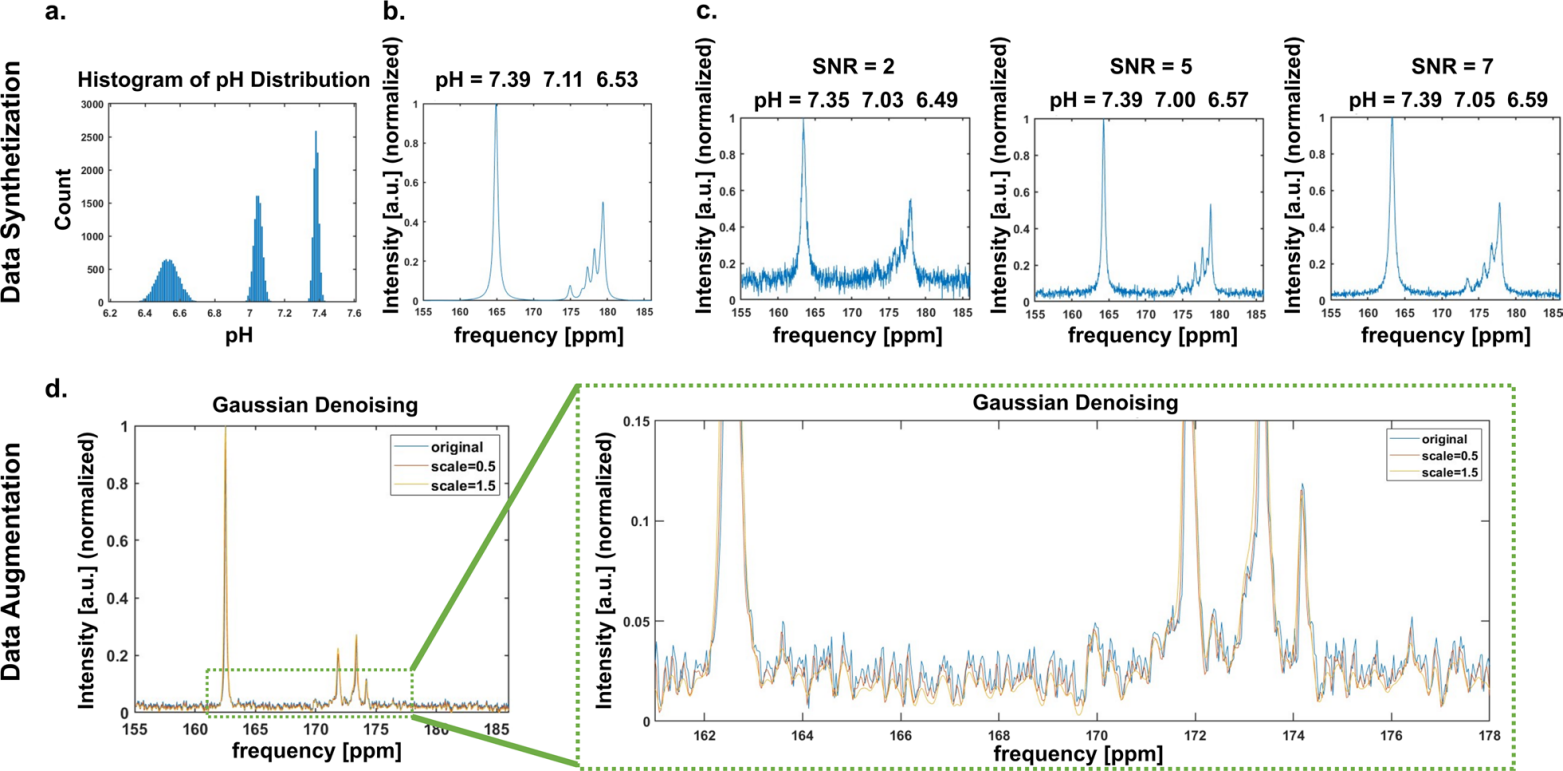
Spectra synthetization was performed to generate three pH_e_ compartment spectra for the CNN and MLP training data. **a** The pH_e_ values for each of the three compartments (7.33–7.44; 6.96–7.15; 6.32–6.78) are normally distributed. **b** An example of the generated spectrum by 3 compartment pH_e_ values. **c** Signal-to-noise (SNR) ratios are applied ranging from 2 to 7 for the synthetic spectra. For simplification, only SNR 2, 5, and 7 are shown. Kidney spectra were augmented by applying five-scale Gaussian denoising to increase the real training data size. **d** Example of the original and denoised spectra. For simplicity, only the first and the fifth scale-denoised spectra are shown. An enlarged version of the spectra (green box) is shown on the right

Due both to the fact that in vivo experiments are necessarily small in sample size for ethical reasons, and that in vivo hyperpolarized ^13^C experiments are labour intensive, synthetic data was generated for the purpose of training the neural networks. To avoid over-fitting, noise was included in the spectral synthetization and was performed based on the following model:

3 $$X\left( f \right) = b_{0} + \varepsilon + S \cdot x\left( f \right)$$

where *X*(*f*) is the synthetic spectrum, *b*_0_ is a constant baseline, $\varepsilon$ is the additive noise, *S* is the SNR scale factor, *x*(*f*) is the set of Lorentzian peaks for the 3 metabolite peaks: urea, ZA_5_ and ZA_1_. Figure 2a shows the distribution of the 3 pH_e_ compartment values of the synthetic spectra, which were initiated from a normal distribution respectively in the ranges: 7.33–7.44; 6.96–7.15; 6.32–6.78, which were found from the measurements carried out according to “Hyperpolarized ^13^C-magnetic resonance spectroscopy” section. The urea peak positions and widths were initiated from a normal distribution with a standard deviation of 0.580 ppm starting from 163 ppm, to represent potential B_0_ inhomogeneities and the peak widths were initiated between 30 and 70 Hz (0.397–0.927 ppm) to account for variations in shim quality. The corresponding chemical shifts ZA_5_ and ZA_1_ peaks were then found by Eq. 2 using the Lorentzian peak model, and a set of basis peaks for the 3 metabolites peaks urea, ZA_5_ and ZA_1_ was then generated (Fig. 2b). The ratios for the urea, ZA_5_ and ZA_1_ peak amplitudes were set to be 4:1:2, which represents the in vivo signal ratios of zymonic acid peaks detected in the different pH_e_ compartments compared to urea. Gaussian noise and baseline were added to the set of combined basis peaks to take the noise from MRS acquisition into account. The range of SNR scale factor was 2 to 7 and baseline was set from − 0.2 to 0.2, both drawn from the normal distribution (Fig. 2c). 10,020 spectra were synthesized, 10,000 spectra were used for training and 20 spectra were used for testing.

##### Kidney data and augmentation

To adapt the neural networks to our target domain of mice kidney spectra, we generated 40 augmented spectra as training data based on the eight acquired mice kidney spectra by applying a five-scale Gaussian denoising (scale factors 1.5, 1.2, 1, 0.8, 0.5) (Fig. 2d). The original eight spectra were used for testing.

#### Experiments

We set up four experiments to evaluate the performance of CNN and MLP on mixed training data: CNN_mix_, MLP_mix_, and single type of training data: CNN_syn_, and MLP_syn_. A total of 10,000 spectra were used (Training: 8500, Validation: 1500). CNN_mix_ and MLP_mix_ consisted of 9960 synthetic spectra, 40 augmented kidney data from PRESS and ROI-averaged CSI acquisitions, whereas CNN_syn_ and MLP_syn_ consisted of 10,000 synthetic datasets. All augmented spectra were set to size of 1024, the magnitude of all spectra was normalized between 0 and 1 for training and testing.

A total of 28 spectra were used for testing, which included 20 augmented spectra and eight real mice kidney spectra. Generation of augmented spectra and pre-processing of in vivo kidney spectra was implemented in MatLab.

#### Network training

Both the CNN and the MLP were trained with a batch size of 200 spectra and 400 epochs. Training progress was achieved by minimizing the sum of mean-square error loss of three pH_e_ compartments: $L = L_{{\text{cortex }}} + L_{{\text{medulla }}} + L_{{{\text{ureter}}}}$, where *L*_*i*_ is the L2 norm of the difference between the predicted pH_e_ compartment from the input spectra, and the ground truth pH_e_ compartment. The loss was then back-propagated for updating the weight kernel for each layer using NADAM (Nesterov-accelerated Adaptive Moment Estimation) optimizer [32]. Both networks were implemented in Keras using TensorFlow as the backend [33]. The training time for both neural networks was approximately five minutes, both training and testing were performed on a NVIDIA Tesla P100 GPU.

### Synthesis of line fitted pH values and neural network predicted pH values into a combined pH map

Based on the network performance results from tests on synthetic and real kidney data, the best performing network is chosen for neural-network-assisted improvement of pH mapping in healthy mice kidneys. For this purpose, supervised line fitting was performed voxel-wise on seven CSI data sets from four mice for which the correct number of fitted compartments and fit quality was assessed. Spectra which were fitted with three pH compartments were extracted. For each image, a segmentation mask was created, indicating voxels either containing three pH compartments (“1”) or less than three compartments (“0”; corresponding to 0, 1 or 2 pH compartments), and fed voxel-wise into the best-performing neural network. pH maps for each CSI data set were then generated where the segmentation mask-positive area pH values were replaced by the predicted pH values from the network for the respective compartment, resulting in hybrid pH maps which are composed of pH values either based on line fitting or neural network predictions. The corresponding mean pH maps were calculated by averaging all compartmental pH values.

## Results

### ^13^C spectroscopic data

Spectra from ^13^C-acquisitions of healthy kidney (top “Input” in Fig. 1) show the urea peak (164 ppm), six zymonic acid peaks (173–178 ppm) and the C_5_-peak of parapyruvate-hydrate (179 ppm), a decay product of zymonic acid. For zymonic acid, three pairs of C_1_ and C_5_ peaks can be grouped unambiguously to a single pH_e_ compartment, and each compartment corresponds respectively to the three anatomical regions of the kidney, namely the cortex (red stars), the medulla (green diamonds) and the ureter (blue triangles). Multiple measurements on three mice return consistent pH_e_ values for the cortex (pH_e_ = 7.38 ± 0.03, *n* = 13), the medulla (pH_e_ = 7.06 ± 0.06, *n* = 11) and the ureter (pH_e_ = 6.53 ± 0.16, *n* = 9).

### Network training

**Fig. 3:**
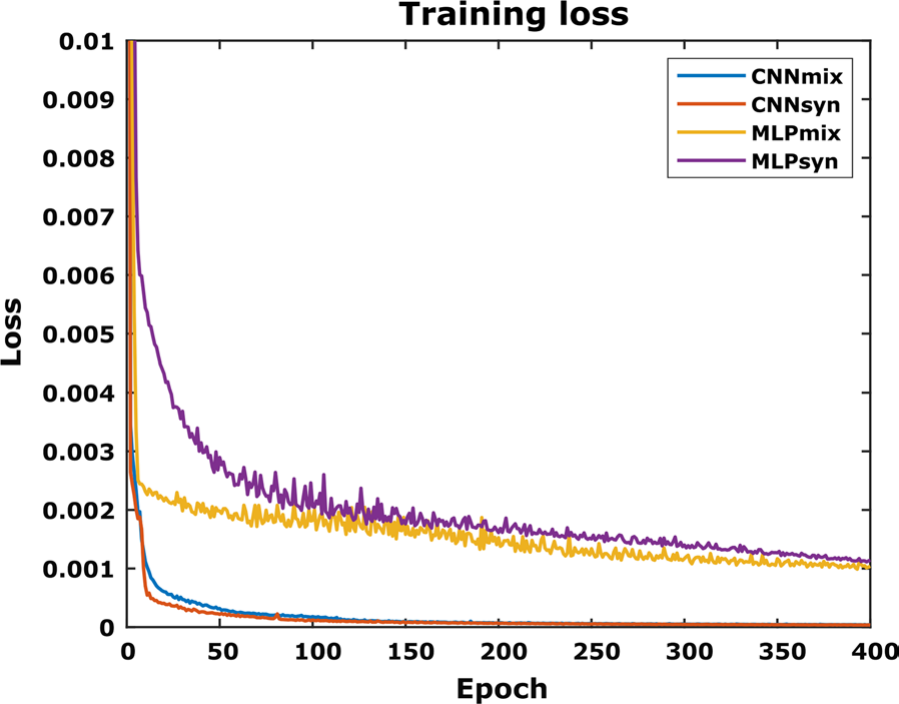
The training losses for four models: CNN_mix_, CNN_syn_, MLP_mix_ and MLP_syn_, stably converge to a minimal loss. While the CNNs achieve absolute minimum loss after 400 epochs, the MLPs remain at a considerably higher loss having not yet reached their absolute minimum at this training stage

The training losses of CNN_mix_, CNN_syn_, MLP_mix_ and MLP_syn_ over 400 epochs are shown in Fig. 3. While the CNNs rapidly converge to their respective limit, the MLPs’ minimal loss after 400 epochs remains higher compared to the CNNs, having not yet reached a converging limit. Interestingly, for both networks, this behaviour is independent of the training data set.

### Comparison of performance of neural networks on augmented test data and conventional line fitting

**Fig. 4:**
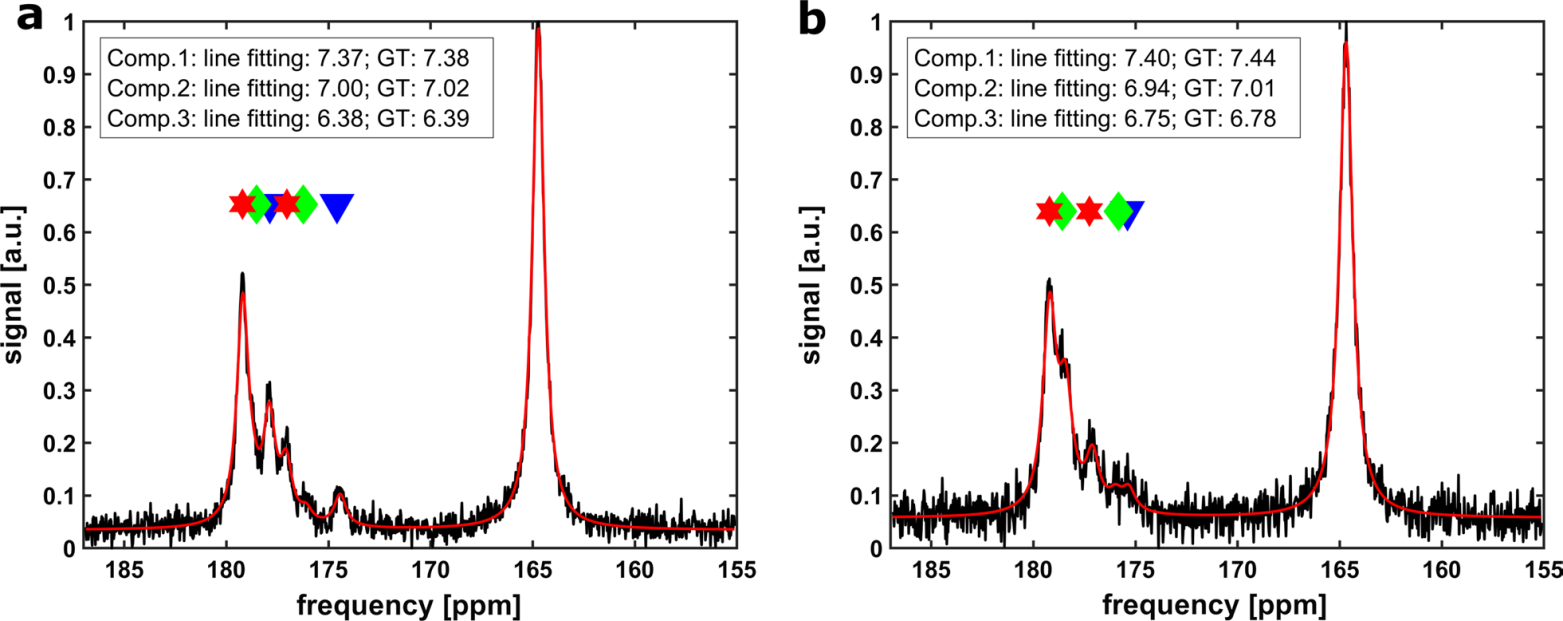
**a** Conventional line fitting of a synthetic kidney spectrum with three fitted pH_e_ compartments indicated by coloured markers (compartment 1: red stars, compartment 2: green diamonds, compartment 3: blue triangles) and comparison of calculated pH_e_ values and ground truth data is shown as inset resulting in good agreement. **b** Conventional line fitting on a noisy spectrum and low compartment intensities of the medulla and the ureter. Three compartments can only be partially detected and agreement with ground truth is rather poor

To validate the network outputs and perform a comparison of the trained networks and conventional line fitting performed by an expert MRS scientist, 20 synthetically generated spectra with known ground truth pH_e_ compartments were blind-fitted by the conventional line fitting and pH analysis routine. In cases of sufficient pH difference between compartments together with sufficient SNR, all peaks can be detected reliably with a high accuracy of the predicted pH_e_ values (Fig. 4a).

**Fig. 5:**
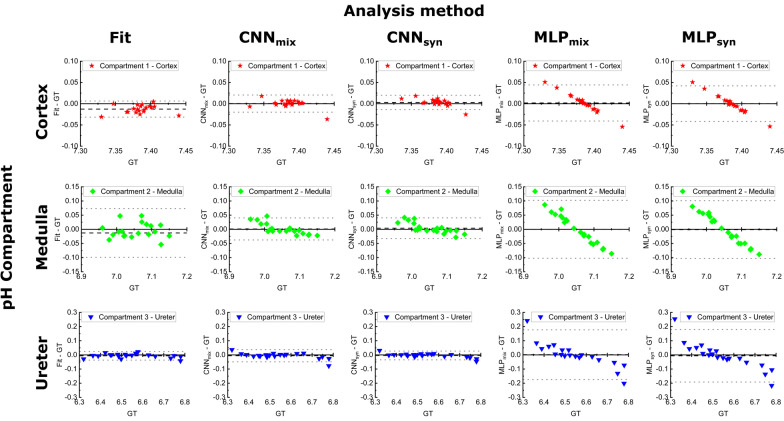
Modified Bland–Altman plots showing the difference between predicted and ground truth pH values from synthetic kidney test spectra against the ground truth pH for each compartment. Black dashed lines indicate the mean difference and grey dotted lines indicate the 95% confidence interval for this deviation

In cases of low SNR and low intensity for one or multiple compartments relative to a third one, the conventional line fitting only partially allows for detection of pH_e_ compartments with less accuracy when comparing to the ground truth pH values (Fig. 4b). In the next step, the same twenty spectra were analyzed with the two CNN models CNN_mix_ and CNN_syn_ and the two MLP models MLP_mix_ and MLP_syn_ which were all previously trained either with a mixed (augmented + synthetic spectra) or with synthetic spectra only. The performance of the conventional line fitting and the four network models on the synthetic spectra test set relative to each other was compared by linear regression of the predicted and the ground truth pH value for each compartment. The adjusted coefficient of determination *R*^2^ and slopes of the linear regression *β* to compare statistical and systematic uncertainties respectively are listed in Table 1. The dominant pH_e_ compartment of the cortex is best predicted by the CNN_mix_ (*β* = 1.01), however with greater uncertainty (*R*^2^ = 0.78) compared to conventional line fitting (*R*^2^ = 0.85) or CNN_syn_ (*R*^2^ = 0.90). The pH_e_ compartment of the medulla is best and equally well predicted by CNN_mix_ and CNN_syn_ compared to all other methods, however with greater systematic uncertainty (*β*_CNNmix_ = 1.30, *β*_CNNsyn_ = 1.26) and lower scattering (*R*_CNNmix_^2^ = 0.91, *R*_CNNsyn_^2^ = 0.92) compared to the cortex. Interestingly, for the pH_e_ compartment of the ureter, line fitting achieves equivalent statistical uncertainty (*R*^2^ = 0.99) compared to the CNNs (*R*_CNNmix_^2^ = 0.98, *R*_CNNsyn_^2^ = 0.99) while outperforming them regarding systematic error (*β*_Fit_ = 1.02). The performance of the MLPs is very poor for the cortex and the medulla compartment and only modest for the ureter compartment. This observation of poor MLP performance is also visualized in the modified Bland–Altman plots (Fig. 5), indicating that the MLPs have deviations of more than 0.2 pH units for the ureter compartments for some spectra. Furthermore, the good performance of both trained CNNs for all compartments is verified while for the conventional line fitting some systematic underestimation of the cortex pH_e_ can be observed.

**Table 1:** Top: Evaluation of the prediction accuracy by compartment-wise comparison of the adjusted coefficient of determination R^2^ derived from a linear regression of ground truth and pH values predicted by conventional line fitting (model “Modelling of NMR pH_e_ spectra” and “Data analysis and conventional line fitting” sections) and the neural networks after application to 20 synthetic test spectra; Bottom: Linear slope coefficients *β* derived from linear regressions to evaluate prediction bias

*R*^2^	Line fitting	CNN_mix_	CNN_syn_	MLP_mix_	MLP_syn_
Compartment 1—Cortex	0.85	0.78	0.90	0.11	0.22
Compartment 2—Medulla	0.65	0.91	0.92	0.05	0.08
Compartment 3—Ureter	0.99	0.98	0.99	0.65	0.59

*β*	Line Fitting	CNN_mix_	CNN_syn_	MLP_mix_	MLP_syn_
Compartment 1—Cortex	0.81	1.01	1.30	3.83	4.79
Compartment 2—Medulla	0.59	1.30	1.26	1.87	2.64
Compartment 3—Ureter	1.02	1.09	1.08	1.52	1.62

Both parameters show poor accuracy and strong prediction bias for the medulla for line fitting and MLP networks potentially due to low SNR

### Comparison of performance of neural networks on real kidney data

**Fig. 6:**
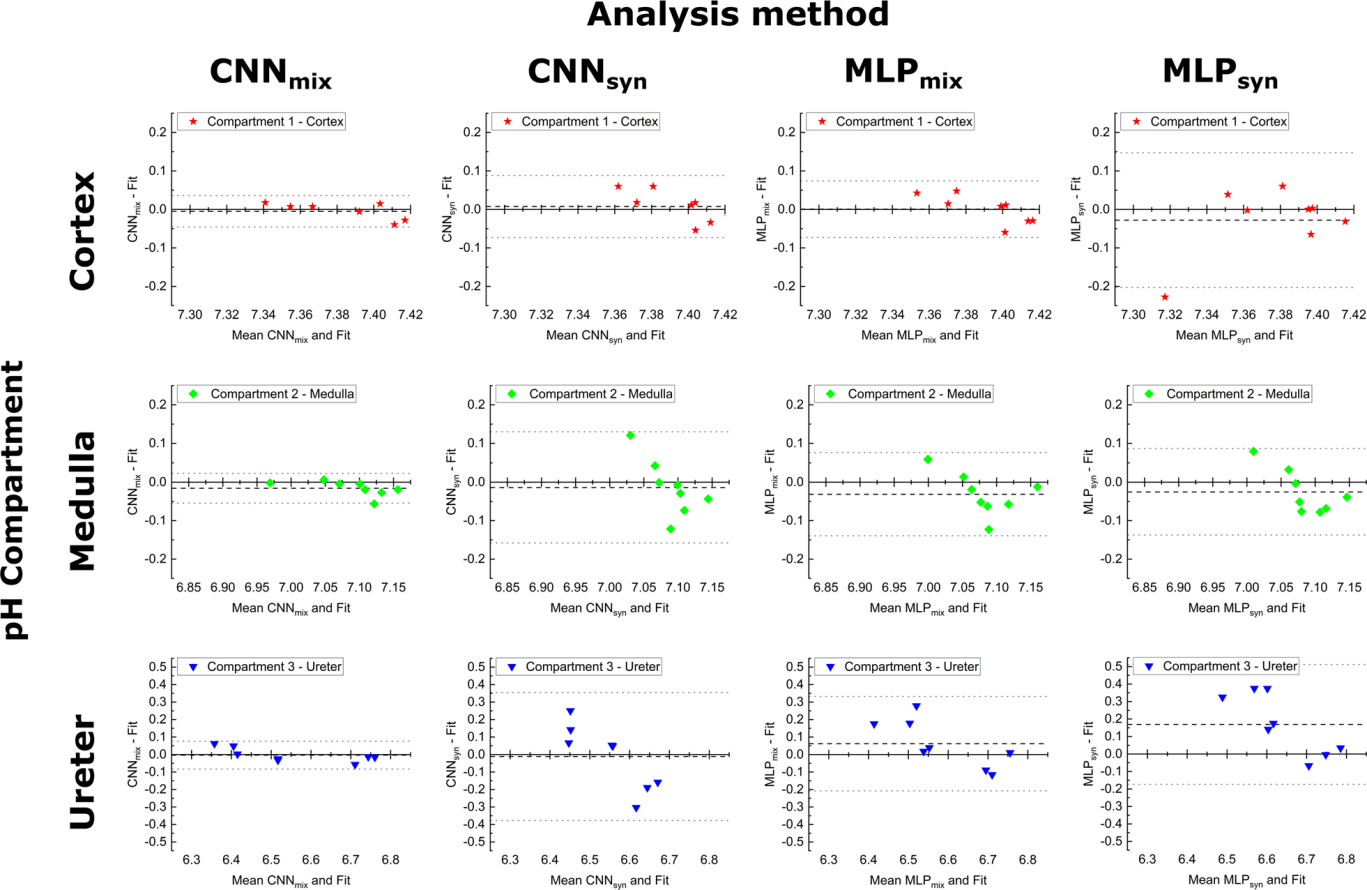
Conventional Bland–Altman plots showing the difference between predicted and conventionally fitted pH_e_ values from spectra of hyperpolarized ^13^C acquisitions on mice kidney plotted against the mean pH_e_ from both analysis methods. Black dashed lines indicate the mean difference and grey dotted lines indicate the 95% confidence interval for this deviation

To evaluate the suitability for routine spectroscopic data analysis, all four networks were tested on eight real mice kidney spectra. A comparison to the values obtained by conventional line fitting as a pseudo ground truth is visualized with Bland–Altman plots (Fig. 6). Following the trend observed for the synthetic test data, the CNN_mix_ network outperforms all other networks, showing the predicted pH_e_ values to deviate less than 0.1 pH units from the fitted data for all compartments. In contrast to testing on synthetic spectra, a CNN network being trained only with synthetic data CNN_syn_, shows only poor performance when tested on real data with predicted pH_e_ values deviating up to 0.3 pH units from conventional fit values and decreasing performance from compartment 1 (cortex) to compartment 3 (ureter). For compartment 2 (medulla) and 3 (ureter), MLP_mix_ also achieves better agreement with fitted pH_e_ values. Analogous to testing on synthetic test spectra, MLP_syn_ shows the worst agreement with conventional line fitting, exceeding 0.1 pH units mean difference for compartment 3 (ureter).

### Hybrid pH mapping by voxel-wise combination of pH values reconstructed from CNN_mix_ and line fitting

**Fig. 7:**
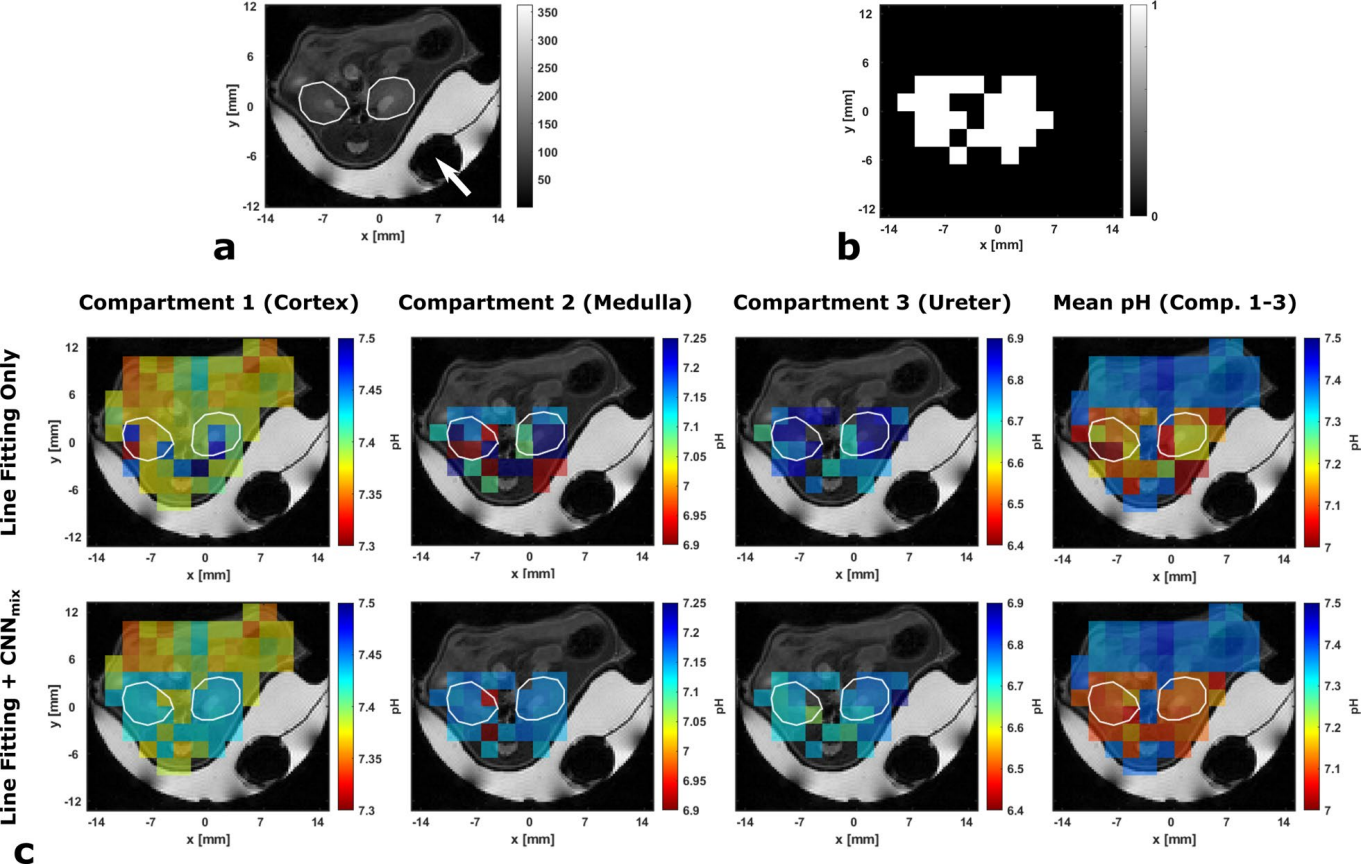
**a** Anatomical axial *T*_2_-weighted image of mice kidney encircled with white ROIs. For shim improvements, the mouse, as well as a [1-^13^C]lactate-phantom for *B*_1_ calibration are covered with carbomer gel. **b** Segmentation mask for a 14 × 12 CSI data sets acquired on the anatomy in **a**. White areas indicate voxels corresponding to spectra with three pH compartments, black areas indicate voxels of which spectra contain two or less compartments. **c** Top row: Individual pH compartment maps for the cortex or a physiological pH compartment (compartment 1), a slightly acidified compartment, mainly the medulla (compartment 2) and a strongly acidified compartment of the ureter (compartment 3) as derived from supervised line fitting. The mean pH map represents the un-weighted average of all three pH compartment maps. Bottom row: Compartment maps derived from line fitting where white areas in the segmentation mask have been replaced by voxel-wise predicted pH values from CNN_mix_. The mean pH map displays the average pH value of the respective number of pH compartments

Based on the performance measurements for synthetic and real kidney spectra, CNN_mix_ was chosen for application in pH mapping of healthy mice kidney (Fig. 7a). An exemplary segmentation mask for CSI data matching the anatomy in Fig. 7a based on supervised line fitting to distinguish voxels with three pH compartments and voxels with less than three pH compartments is shown in Fig. 7b (white: three pH compartments, black: less than three pH compartments).

pH mapping based on supervised line fitting (Fig. 7c, top row) reveals a globally present physiological pH compartment (top left), a heterogeneous, slightly acidic second pH compartment which can be attributed to the medulla (top middle-left) and a third pH compartment corresponding to the ureter (top middle-right). Voxel-wise compartment-averaging generates kidney-specific pH contrast. Substitution of line fitted pH values by values predicted by CNN_mix_ shows slightly more basic pH values predicted by the neural network compared to the line fitting. For the medulla compartment, network predictions appear to be more homogeneous compared to line fitted maps. For the ureter compartment, line fitted as well as neural network predicted maps agree well with each other. The mean pH map based on these hybrid compartment pH maps shows good inter- and intra-kidney homogeneity in mean pH values compared to line fitted maps.

**Fig. 8:**
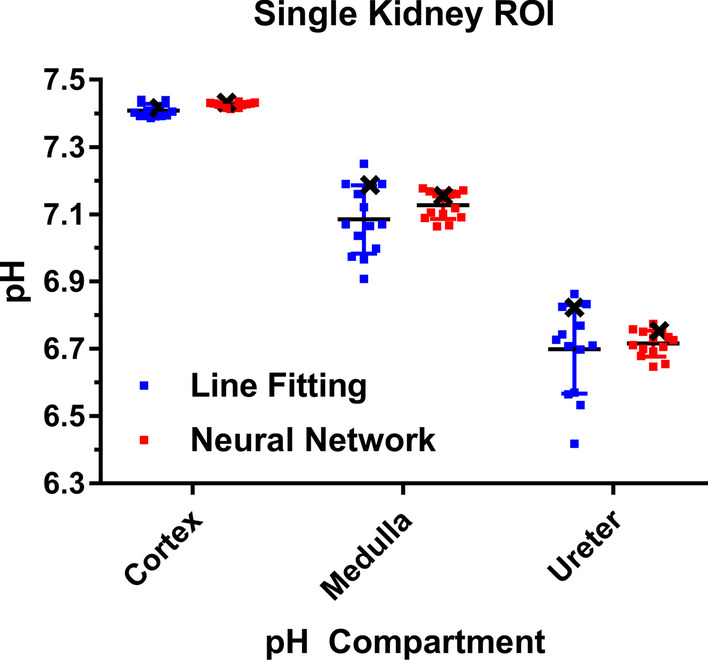
pH compartments averaged over regions of interests in single kidneys of mice. pH values derived from line fitting show stronger variations between single kidneys and different subjects compared to predictions from CNN_mix_.pH values for each compartment show good agreement between line fitting and neural network predictions for individual kidneys (example indicated by black crosses)

For quantitative comparison, pH compartments derived from line fitting and neural networks were both averaged across individual kidneys for multiple acquisitions on different animals (Fig. 8). pH values derived from line fitting show lowest inter- and intra-subject variation for the cortex (pH_cortex,fit_ = 7.41 ± 0.02, *n* = 14) while pH values for the medulla (pH_medulla,fit_ = 7.09 ± 0.10, *n* = 14) and the ureter (pH_ureter,fit_ = 6.70 ± 0.13, *n* = 14) are distributed across larger pH ranges while all compartments can be well separated from each other based on pH. pH compartments predicted by the CNN_mix_ agree well with line-fitted compartments, despite the cortex (pH_cortex,CNN_ = 7.43 ± 0.01, *n* = 14) and the medulla (pH_medulla,CNN_ = 7.13 ± 0.04, *n* = 14) exhibiting overall slightly more basic pH values compared to the line-fitted ones. For the ureter, no relevant difference can be observed (pH_ureter,CNN_ = 6.72 ± 0.04, *n* = 14). In agreement with lower intra-subject variations as seen in compartment maps in Fig. 7, the inter- and intra-subject variations of compartment pH values are lower for the neural-network-predicted pH values, while the values for each subject are in good agreement relative to the compartment-specific standard deviation (black crosses are corresponding to the same kidney in the same subject in Fig. 8).

## Discussion

### Modelling and fitting of hyperpolarized ^13^C spectroscopic data

Analogous to published data on spectra of zymonic acid in kidney of healthy rats [13], several pH compartments can be detected in hyperpolarized ^13^C acquisitions of zymonic acid in healthy kidney of mice. However, out of three detected pH_e_ compartments which could be assigned to cortex, medulla and ureter, the latter two often suffer from lower compartment signal due to limited perfusion of the agent within the short acquisition time [13] or enhanced signal decay in case of injury or pathological alterations [34–36]. Consequently, line fitting with conventional methods becomes challenging when trying to resolve the pH_e_ compartments of the kidney. For these reasons, manual spectral pre-processing together with fitting of a linear combination of individual Lorentzian peaks while minimizing the free parameters to amplitude, peak position, and peak width (FWHM) was chosen as an appropriate fitting routine despite inherent SNR limitations. For these reasons, manual spectral pre-processing together with fitting of a linear combination of individual Lorentzian peaks while minimizing the free parameters to amplitude, peak position, and peak width (FWHM) was chosen as an appropriate fitting routine despite inherent SNR limitations. Additionally, other standard fitting routines for MR spectra such as LCModel [37] or AMARES [38], were either unsuitable, unstable, or of no significant benefit in this work.

For instance, for AMARES it was already shown that this algorithm suffers from unstable fitting when the peak frequencies are allowed as free parameters [39]. In addition, zymonic acid spectra on kidney are less sparse than the previously fitted pyruvate- and lactate-containing spectra.

LCModel is a standard fitting routine for magnetic resonance spectra which allows excellent peak quantification for ^1^H metabolites. Nevertheless, this method also has several limitations regarding the application to the data of this work. LCModel predominantly aims to quantify spectral peaks which requires the input of a set of basis spectra of high spectral quality and good SNR. In such cases, the peak positions are fixed, and only minor peak shifts due to eddy currents and magnetic field inhomogeneities are tolerated. This contrasts with hyperpolarized ^13^C acquisitions using zymonic acid in which SNR is typically modest, spectral resolution limited and quantification not necessary. As zymonic acid peaks strongly shift with pH_e_, a suitable set of basis spectra would require multiple zymonic acid spectra at different pH_e_ values which either requires a high amount of basis components or an inherent limitation in the measured pH accuracy limited by a small basis set. In addition, since C_1_- and C_5_-peak intensities vary relative to each other in different acquisitions, combined modelling as one basis spectrum for a fixed pH is difficult. Furthermore, LCModel requires well separated peaks for proper differentiation which is not the case for the densely packed pH compartment peaks as seen in Fig. 1.

### CNN and MLP performance

In our study, both CNN_mix_ and CNN_syn_ outperform MLP_mix_ and MLP_syn_ in predicting all three pH compartments in synthetic test data. Here, the CNNs have better accuracy and less uncertainty as shown in the regression analysis. Interestingly, for the cortex and medulla compartments, CNN_mix_ was giving a better prediction accuracy as compared to the conventional line fitting method. While the CNN and the MLP have a similar number of weights (≈ 8000), the CNN used kernels in convolutional layers to perform elementwise multiplications to inputs while the MLP used densely connected neurons. When applied systematically across the entire input spectra, these convolutional kernels could extract spectral features such as the metabolite peaks distances, as the kernels account for the values on neighboring pixels. Because of the weight sharing that occurs when the convolutional kernels slide across the spectrum [40, 41], the CNN becomes less susceptible than the MLP to spectral variance or drifts in spectral peak positions which can be caused by *B*_0_ inhomogeneities. However, the choice of specific neural network depends on the type of learning tasks and features to be extracted, as previous studies showed MLP performed well in classification [42, 43], while CNN also demonstrated good performance in image segmentation [44] or classifications [45]. Some even explore the synergies of MLP and CNN networks [46, 47].

### Performance of neural networks trained with mixed data

We showed that it is possible to train models on a limited amount of real data by transfer learning, whilst most of the training data were synthetically generated based on a spectral model for [1,5-^13^C_2_]zymonic acid and ^13^C-urea. When tested with synthetic data, CNN_syn_ performed better than CNN_mix_ in predicting medulla and ureter pH as shown in the linear regression analysis and modified Bland–Altman plots. Especially for the medulla compartment, both line fitting and MLP_syn_ and MLP_mix_ show poor performance what might be due to higher sensitivity to low SNR. As there is no absolute ground truth for the kidney in healthy mice, we compared the neural network predictions with the results in line fitting as a pseudo ground truth. We found CNN_mix_ had the most consistent and comparable results to line fitting, as it has the smallest difference compared to other models, and its 95% confidence level is also smaller than CNN_syn_ in cortex and ureter (see Fig. 5). Moreover, MLP_mix_ are more comparable to line fitting than CNN_syn_, and MLP_mix_ has generally smaller mean difference.

### Hybrid pH map generation by combination of line fitting and CNN_mix_ predictions

Combination of line fitting and neural networks appears to improve pH mapping in kidneys of healthy mice. Based on compartmental pH maps, line fitting appears to be only quantitatively robust for the cortex while the medulla and the ureter show considerable inter- and intra-kidney pH variability, the latter being physiologically rather unreasonable. We assume that this high variability stems from the low SNR of zymonic acid peaks corresponding to these two compartments. Substitution of voxels in pH maps corresponding to spectra containing three pH compartments by neural network predictions results in more homogeneous compartment maps while quantitatively still agreeing with line-fitted compartments. This suggests a superior performance of the neural network compared to the line fitting approach for low SNR compartments. Furthermore, as the network is predicting pH compartments voxel-wise based on individual spectra, it has to be pointed out that the predictions of neighboring voxels are independent from each other and therefore the observed spatial homogeneity of compartments therefore indicates a good robustness of the pH predictions. In addition, high quantitative prediction accuracy is suggested by the observation that cortex compartments are systematically predicted with a higher pH value compared to the line fitting method, which agrees with the observation that line fitting systematically underestimates the cortex pH when evaluating the method performance for artificial spectra of known pH.

### Data size

These observations suggest that the real and augmented data might consist of spatially independent features, such as the noise during the spectra acquisition, which is crucial to train a more accurate model. Neural networks usually require a large amount of training data, and the number of training data depends on the complexity of the tasks and features to extract. However, generating a larger data set is challenging for hyperpolarized ^13^C MRSI. In vivo spectra obtained by preclinical studies are limited in size for ethical reasons: the number of animals should be as low as possible. Additionally, the experimental efforts are rather large. Also, with regard to application for hyperpolarized ^13^C acquisitions in humans, data set size is critical as clinical trials currently performed with this imaging technique are typically limited to 5–100 patients [48]. Efforts to obtain larger amounts of data might involve the generation of databases but, especially for imaging using hyperpolarized ^13^C-labelled zymonic acid, this is at an early stage. Nevertheless, in our study, we showed improved network performance by including less than 0.5% real augmented data (40 augmented spectra out of 10,000 training spectra), an amount that can be realistically generated from single preclinical studies.

### Future works

In this study, we found that the convolutional layers enable the network to better extract spectral features in the spectra. Future works could extend the application of convolutional layers to denoise the spectra or automating peak picking. Moreover, the neural networks here only predict the chemical shift of the spectra—they do not yet consider the signal intensity as in the conventional line fitting method. An extension the current approach might also predict signal intensity, which could then allow a more direct calculation of weighted-average pH maps. In addition, for imaging of cancer or unknown tissue, networks could be trained to predict the correct number of pH compartments and using this information to selectively pass the spectra through other networks which predict the correct pH values.

## Conclusion

Two different types of neural networks trained once with a fully synthetic data set and once with a mixed data set, containing real and synthetic data, were each evaluated for prediction of pH compartments from hyperpolarized ^13^C acquisitions of zymonic acid on kidney in healthy mice. CNNs trained with a mixed set of augmented and synthetic spectra show the ability to accurately predict multiple pH compartments in hyperpolarized ^13^C spectra. This network achieves the best results out of all tested networks and its performance competes with or outperforms conventional line fitting being supervised by humans. The trained network can be used to improve pH mapping by segmentation-based substitution of line fitted pH values by neural network predictions. Therefore, small amounts of experimental data and appropriate neural network and training method choice allows fast, accurate, and reliable evaluation of hyperpolarized ^13^C magnetic resonance spectroscopic acquisitions for pH measurements in kidney. Using appropriate training data sets and slightly modified output layers of the networks to account for different amounts of detected pH compartments, the presented concept could potentially be applied to other organs or tumours.

## Data Availability

The data sets used and analyzed during the current study are available at https://github.com/ryanayf/KidNeYronal
